# The Possible Efficacy of Artichoke in Fluconazole Related Hepatotoxicity

**DOI:** 10.1155/2014/697359

**Published:** 2014-09-03

**Authors:** Hüseyin Kurt, Omer Toprak, Erdoğan Bülbül

**Affiliations:** ^1^Department of Internal Medicine, Balıkesir University School of Medicine, 10020 Balıkesir, Turkey; ^2^Department of Radiology, Balıkesir University School of Medicine, 10020 Balıkesir, Turkey

## Abstract

Although fluconazole related hepatotoxicity (FRH) is rare, mortal acute hepatic necrosis and jaundice were reported in immunocompromised states such as acquired immunodeficiency syndrome (AIDS) and bone marrow transplant (BMT). We present a case of a patient with multiple sclerosis who developed hepatotoxicity with the use of a single 150 mg fluconazole tablet for fungal vaginitis, 10 days after methylprednisolone pulse treatment. Our patient's alanine aminotransferase (ALT) and aspartate aminotransferase (AST) levels were decreased, 1200 U/L and 800 U/L, respectively, and bilirubin levels were consistent at 37 mg/dL. Artichoke which has anticholestatic and antioxidant properties was used by our patient. She consumed a 30 mg artichoke leaf extract tea 3 times a day. The bilirubin levels significantly declined at the end of the first week and all liver function tests were normalized within 2 months.

## 1. Introduction

Fluconazole is a member of new triazole antifungal family and it may rarely cause hepatotoxicity and sudden death in patients with severe medical handicaps [1]. In daily use it was frequently prescribed by gynecologists, dermatologists, and infection specialists. The most common side effects are nausea, diarrhea, abdominal pain, and distention and skin rashes. The fluconazole related hepatotoxicity (FRH) is mostly reversible with the discontinuation of the drug. In this paper, for the first time, we present reversal of FRH related to pulse steroid treatment in a patient who consumed an artichoke dominant diet.

## 2. Case Report

**Figure 1:**
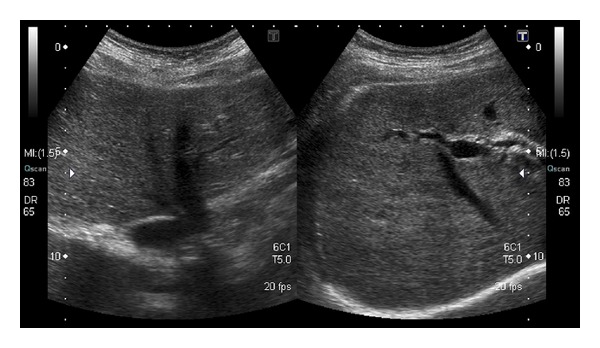
Abdominal ultrasonography (coarsening in liver parenchyma and minimally prominent intrahepatic bile ducts).

A 40 year old female medical doctor with 18-year history of multiple sclerosis had received 1 g/day pulse methylprednisolone for 10 days following her last acute attack. The patient had not used any type of maintenance therapy for multiple sclerosis. 10 days after the pulse steroid treatment, the patient consumed a single dose of 150 mg fluconazole oral tablet for the treatment of vaginitis. On the 5th day after usage the patient developed nausea, on the tenth day tiredness and darkening of the urine, and icterus in sclera and skin on the 12th day. On admittance to internal medicine and gastroenterology department her labs were as follows: alanine aminotransferase (ALT) = 1180 U/L, aspartate aminotransferase (AST) = 800 U/L, gamma-glutamyltransferase (GGT) = 81 U/L, total bilirubin = 6.4 mg/dL, direct bilirubin = 4.6 mg/dL, INR = 1.38, and alkaline phosphatase (ALP) = 85 U/L (Figures 1(a) and 1(b)). Complete blood count, blood urea nitrogen, and serum glucose were normal. Bilirubin and urobilinogen were detected in dark and cloudy urine. The serology marker results were normal as follows: HBsAg, anti-HBc IgM and IgG, anti HCV (−), anti-HBs (+), IgM for anti-HAV, CMV, EBV (−), IgG for anti-HAV, CMV, and EBV (+).

Abdominal ultrasound examination revealed minimal coarsening of the liver parenchyma, increased echogenicity in the periportal space, and minimally prominent intrahepatic bile ducts (Figure 1). Autoimmune hepatitis markers antinuclear antibody (ANA), antismooth muscle antibody (ASMA), and antimitochondrial antibody (AMA) were negative. Albumin and globulin levels were normal on protein electrophoresis. The case was accepted as FRH. ALT and AST had increased to 1275 U/L and 907 U/L, respectively; 3 days later and the patient was followed up on an outpatient basis as she rejected hospital admittance. The patient was advised to limit fat and carbohydrate consumption and limit her physical activity.

**Figure 2:**
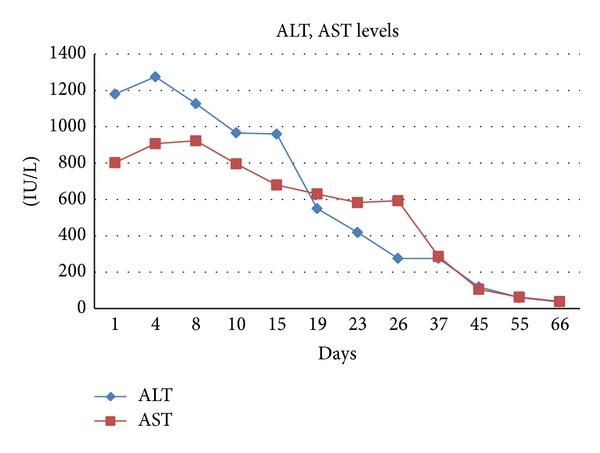
Changings of ALT and AST levels.

**Figure 3:**
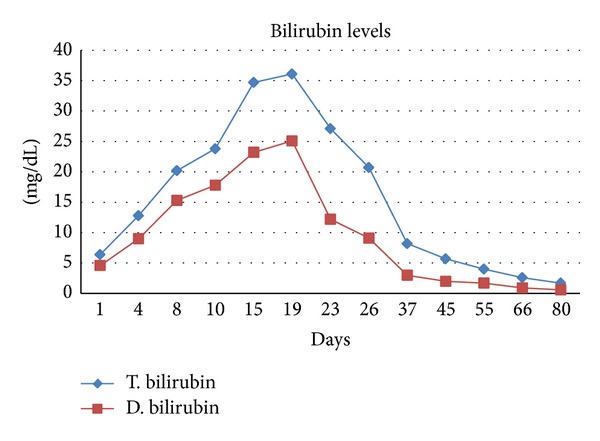
Changings of bilirubin levels.

After 2 weeks, ALT and AST levels decreased to around 100 U/L, whereas total bilirubin increased to 36 mg/dL (Figures 2 and 3). Although the patient's ALT and AST levels decreased, the bilirubin levels remained high and the patient was informed about the possible need for a liver transplant. The patient performed a literature search and found that artichoke had ameliorative effects in liver diseases. Turkish traditional beliefs also support the theory that artichokes have such an effect.

The patient without our knowledge decided to consume artichoke based on both her literature search and the traditional belief. At the follow-up period, the patient had consumed artichoke tea made up of approximately 30 gr of dry artichoke leaves (Enginar Bitki Çayı; Süzen Demlik Poşet, İstanbul, Turkey) 3 per day (1.5 gr/kg body weight) [2]. Artichoke leaf extract was used by the permission of the patient. One week later, total bilirubin level decreased from 36 mg/dL to 27 mg/dL and ALT-AST levels normalized. The decrement continued until the levels normalized 2 months after the initial diagnosis.

## 3. Discussion

Fluconazole acute hepatotoxicity is rare compared to other antifungal agents [3]. Serum ALT and AST levels may be elevated in 5–10% of patients using fluconazole but most of the time patients remain asymptomatic and continue their treatment [4]. The 8-fold increment of ALT levels that necessitates the withdrawal of the fluconazole treatment is encountered in 1% of the patients [4–6]. Although FRH incidence is low, severe jaundice, fatal acute hepatic necrosis, and even death have been reported in patients with acquired immunodeficiency syndrome (AIDS) and bone marrow transplant (BMT) [1, 7]. Methylprednisolone treatment also causes immunosuppression like AIDS and BMT. Our patient used methylprednisolone 1 gr per day for 10 days. A recent study demonstrated that fluconazole was responsible for 12 of 52 antifungal related hepatotoxicity cases and all 6 patients who died in this study were fluconazole users [8].

Fluconazole is not metabolized in the liver and is excreted without any change through the kidneys. Dose related hepatotoxicity for this reason might be seen in the elderly and kidney failure patients due to their limited kidney function. Therefore, it should be used with precaution in this subset of patients [8, 9]. Our patient was neither old nor did she have abnormal kidney function.

Drug related liver damage has 3 characteristics; there should be no concurrent liver disease, the condition has to regress after the withdrawal of the medication, and the relation between the medication and the condition has to be temporary [10]. In our case the lack of any liver disease and the normalization of ALT, AST, and bilirubin levels after the withdrawal of the drug supported the diagnosis of FRH. A liver biopsy was not performed due to the refusal by the patient. If performed typical findings of fluconazole related hepatotoxicity including portal and lobular inflammation with cholestasis and apoptosis might be documented [11].

High-dose intravenous methylprednisolone treatment is the preferred treatment in MS patients [12]. It is also known that the possibility of fungal infection after the methylprednisolone treatment is increased. Our patient similarly developed fungal vaginitis after methylprednisolone treatment and had faced FRH due to the use of a single dose of oral fluconazole 10 days after the methylprednisolone treatment.

Contrarily, in a study of current literature we encountered a FRH case that was treated with 120 mg/day of methylprednisolone [11]. However it should be noted that the most important part of treatment is withdrawal of the medication.

Artichoke has antioxidant properties via increasing glutathione levels and glutathione peroxidase activities. It is considered as a beneficial agent in oxidative stress-induced hepatotoxicity [2]. Our patient consumed 1.5 gr/kg per day of artichoke leaf extract in a day which was the dose suggested in the literature.

Artichoke is also used for its strong choleretic activity causing substantial increase in the amount of bile excreted and an increase in the biliary acids concentrations of the bile [13]. In vitro investigations had shown that the cynarin, chlorogenic acid, and caffeoyl derivatives present in artichoke and protect hepatocytes against hepatic damage induced by carbon tetrachloride in primary cultured hepatocytes. Its therapeutic activities are mostly attributed to the cynarin content [14].

In our case although ALT and AST levels decreased bilirubin persisted with high levels. That is why we think that the artichoke leaf extracts used in our case exerted its main action with anticholestasis since the refractory high bilirubin levels decreased after its use. This effect might contribute to the overall hepatoprotective influence of this herbal formulation [15].

In our case, although ALT-AST levels have a tendency to decline, bilirubin levels remained high which makes the surgeons consider the possibility of transplantation surgery. The patient who is a healthcare specialist performed a literature search and found that artichoke has favorable results in animal models of FRH. After the consumption of artichoke by our patient, the bilirubin level has started to decline. The decrement of bilirubin might be both related to the natural course of the disease with decrement during the follow up or to the ameliorative effect of artichoke. This point can only be distinguished by prospective randomized trials, which is very hard to perform.

In conclusion, similar to immunosuppression the possibility of FRH and even death after steroid treatment should be kept in mind. Artichoke consumption in FRH might be helpful both with its antioxidant characteristics and with anticholestatic action in cases with higher bilirubin levels.
